# Adenosine Monophosphate (AMP)-Activated Protein Kinase: A New Target for Nutraceutical Compounds

**DOI:** 10.3390/ijms18020288

**Published:** 2017-01-29

**Authors:** Fabiola Marín-Aguilar, Luis E. Pavillard, Francesca Giampieri, Pedro Bullón, Mario D. Cordero

**Affiliations:** 1 Research Laboratory, Oral Medicine Department, University of Sevilla, Sevilla 41009, Spain; fabiolamag@gmail.com (F.M.-A.); luchopavillard@gmail.com (L.E.P.); pbullon@us.es (P.B.); 2 Dipartimento di Scienze Cliniche Specialistiche ed Odontostomatologiche—Sez. Biochimica, Università Politecnica delle Marche, Ancona 60100, Italy; f.giampieri@univpm.it

**Keywords:** adenosine monophosphate-activated protein kinase (AMPK), nutraceutical compounds, cancer, type II diabetes, neurodegenerative diseases, cardiovascular diseases

## Abstract

Adenosine monophosphate-activated protein kinase (AMPK) is an important energy sensor which is activated by increases in adenosine monophosphate (AMP)/adenosine triphosphate (ATP) ratio and/or adenosine diphosphate (ADP)/ATP ratio, and increases different metabolic pathways such as fatty acid oxidation, glucose transport and mitochondrial biogenesis. In this sense, AMPK maintains cellular energy homeostasis by induction of catabolism and inhibition of ATP-consuming biosynthetic pathways to preserve ATP levels. Several studies indicate a reduction of AMPK sensitivity to cellular stress during aging and this could impair the downstream signaling and the maintenance of the cellular energy balance and the stress resistance. However, several diseases have been related with an AMPK dysfunction. Alterations in AMPK signaling decrease mitochondrial biogenesis, increase cellular stress and induce inflammation, which are typical events of the aging process and have been associated to several pathological processes. In this sense, in the last few years AMPK has been identified as a very interesting target and different nutraceutical compounds are being studied for an interesting potential effect on AMPK induction. In this review, we will evaluate the interaction of the different nutraceutical compounds to induce the AMPK phosphorylation and the applications in diseases such as cancer, type II diabetes, neurodegenerative diseases or cardiovascular diseases.

## 1. Introduction

**Figure 1:**
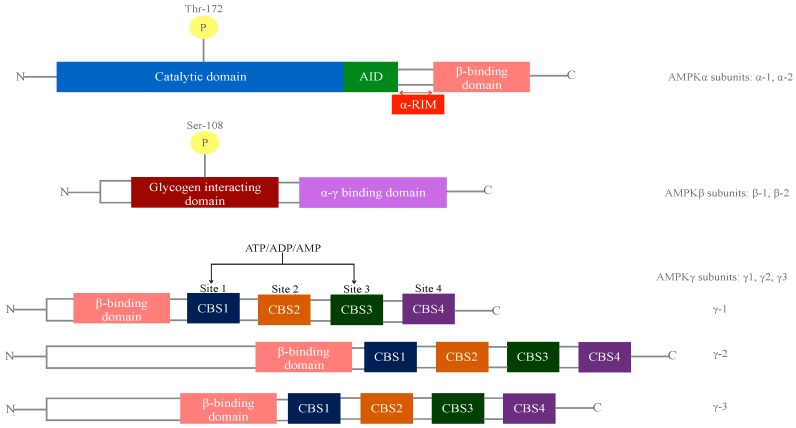
Diagram of the adenosine monophosphate-activated protein kinase (AMPK) domain structure. Two α subunits, two β and three γ subunits have been described to date. The α-subunit is conformed by a catalytic domain containing Thr172 kinase for the activation by upstream kinases, Liver Kinase B1 (LKB1) and Ca^2+^/calmodulin-dependent protein kinase kinase-β (CaMKKb), an auto-inhibitory domain (AID), two regulatory interacting motifs (α-RIMs), and a C-terminal domain that firmly binds to β and γ subunits. The β-subunit contains a N-terminal domain rich in glycine, a carbohydrate binding module (CBM) containing Ser108, important for some direct activators of AMPK, and a C-terminal domain that attaches to α and γ subunits. The γ-subunit consist of three γ isoforms and variable length N-terminal domains and four cystathionine β-synthase domains (CBS) forming bateman domains that create adenosine monophosphate (AMP)/ adenosine diphosphate (ADP)/adenosine triphosphate (ATP) binding sites. All amino acid numbers refer to human AMPK sequences.

Adenosine monophosphate-activated protein kinase (AMPK), is a heterotrimeric protein kinase consisting of an alpha (α) catalytic subunit in combination with scaffolding beta (β) and regulatory gamma (γ) subunits (Figure 1). These subunits, encoded by seven genes: Protein Kinase AMP-Activated Catalytic Subunit α1 (*PRKAA1*), Protein Kinase AMP-Activated Catalytic Subunit α2 (*PRKAA2*), Protein Kinase AMP-Activated Non-Catalytic Subunit β1 (*PRKAB1*), Protein Kinase AMP-Activated Non-Catalytic Subunit β2 (*PRKAB2*), Protein Kinase AMP-Activated Non-Catalytic Subunit γ1 (*PRKAG1*), Protein Kinase AMP-Activated Non-Catalytic Subunit γ2 (*PRKAG2*), Protein Kinase AMP-Activated Non-Catalytic Subunit γ3 (*PRKAG3*) can theoretically combine to form twelve different possible isoforms that may differ in tissue-specific expression and activation. AMPK is known as the fuel of the cell, working to ensure that adenosine triphosphate (ATP) levels are maintained under energetic stress situations such as exercise, starvation, hypoxia or rapid cell growth [1].

The co-expression of the three different subunits (α, β, and γ) of AMPK is absolutely necessary to generate a functionally active protein. The β-subunit is the smallest among the three and serves as a scaffold for anchoring α and γ-subunits by its C-terminal module, conforming the “AMPK regulatory core”. The carbohydrate-binding module (CBM) of the β-subunits sits above the protein kinase module of the α-subunit. The interface between both creates a novel allosteric binding site, recently described as the “ADaM" (allosteric drug and metabolite) site, binding some of the known synthetic AMPK activators [1]. The catalytic domain on the α-subunit (Thr172) is followed by an auto-inhibitory domain (AID) and regulatory interacting motifs (α-RIMS), which are flexible regulatory segments triggering conformational changes in response to adenosine monophosphate (AMP) binding to the AMPK γ-subunit. γ-subunits contain four tandem cystathionine β synthase domains (CBS´s), which are known as bateman domains. γ1, γ2 and γ3 contain four potential nucleotide binding sites, mainly CBS1 and CBS3, known to bind AMP, adenosine diphosphate (ADP) or ATP, inducing a conformational switch that allosterically activates AMPK as well as protecting pTr172 in α- subunits from dephophosrylation by phosphatases [2].

### 1.1. Mechanism of AMPK Activation

As discussed above, AMPK is regulated both allosterically and by post-translational modifications. There are different well-defined mechanisms of AMPK activation by which several small drugs have been developed to induce AMPK phosphorylation. The direct phosphorylation at a kinase site, for example, Thr172 of the α-subunit by upstream kinases Liver Kinase B1 (LKB1) and Ca^2+^/calmodulin-dependent protein kinase-β (CaMKKb) or, Ser108 of the β-subunit, is the mechanism used by salicylate and A769662. Another small-molecule allosteric activator of AMPK is compound 2, C2 (5-(5-hydroxyl-isoxazol-3-yl)-furan-2-phosphonic acid). It is thought that C2 activates both AMPK α1 and α2 isoforms by binding to the γ-subunit. This is a new AMP-mimetic compound similar to 5-aminoimidazole-4-carboxamide ribonucleoside (AICAR), a nucleoside that is taken up into cells by adenosine transporters, both are prodrugs converted into AMP analogs inside cells. Finally, AMP and/or ADP binding to nucleotide binding sites on C-terminal modules of γ-subunits induced conformational changes to protect AMPK from dephosphorylation or inactivation by phosphatases, which suggests that AMPK is a sensor of AMP/ATP or ADP/ATP ratios [1,2].

In order to preserve body energy homeostasis, AMPK, once activated, phosphorylates to activate the pathways corraleted to ATP production, such as glucose transport in muscle via the tre-2/Bub2/Cdc16 domain family member (TBC1D1), fatty acid oxidation via acetyl-CoA carboxylase (ACC2), or autophagy via Unc-51 Like Autophagy Activating Kinase 1 (ULK1). Simultaneously, AMPK inhibits the pathways involved in ATP consumption, for example cholesterol via 3-hydroxy-3methyl-glutaryl-coenzyme A (HMG-CoA) reductase, fatty acid synthesis (ACC1) and protein synthesis (mammalian target of rapamycin complex 1, mTORC1) [1]. Apart from the regulation of metabolic enzymes, AMPK is involved in long-term adaptive changes through the regulation of co-activators (peroxisome proliferator-activated receptor γ coactivator 1-α, PGC1α) and transcription factors (histone deacetylase, HDAC). Importantly, AMPK regulates the activity of another serine/threonine protein kinase, mammalian target of rapamycin (mTOR) [3], enabling cells to react to metabolic stress as well as to regulate autophagy and protein translation through its effects on tuberous sclerosis complex 2 (TSC2) and raptor.

AMPK also plays a crucial role in the regulation of mitochondrial homeostasis, controlling major steps in mitochondrial biogenesis and degradation. It has been demonstrated that AMPK is directly connected with the process of mitophagy, by which it recycles essential nutrients from dysfunctional mitocondria. When this occurs, oxidative damage induced by mitochondria poisons [4] indirectly activates AMPK leading to the phosphorylation of mitochondrial fission factor (MFF) protein, which triggers mitochondrial fragmentation, and in turn, mitophagy. Many naturally-occurring compounds, such as resveratrol or quercetin, are assumed to activate AMPK through this indirect process. They induce mitochondrial dysfunction through inhibiting mitochondrial ATP production and increasing cellular AMP or ADP, indirectly activating AMPK. However, it should be noted that AMPK is usually activated following one of the mechanisms below mentioned [5]:

1. Activation through the AMPK γ-subunit: Prodrugs that are converted into AMP analogs following cellular uptake by intracellular enzymes, such as 5-aminoimidazole-4-carboxamide riboside (AICAR). Upon entry the cell, AICAR is phosphorylated to generate Phosphorylated AICAriboside (ZMP), which works as an AMP mimetic. Another prodrug is compound 13 (C13), that generates isoxazole or C2, a potent activator and protector of α isoforms from dephosforylation.
2. Direct activators binding between the β-CBM domain and kinase domain Ser108. Examples of these AMPK activating compounds are thienopyridone (A-769662), salicylate and 911 compound.
3. A number of agents (metformin or hydrogen peroxide) and natural occurring products (berberin or resveratrol) act as mitochondria poisons (above mentioned) increasing the AMP:ATP ratio or energy charge and therefore activating AMPK indirectly.

AMPK can reach near-activation by any modulator or compound causing AMP accumulation. Whereas indirect activators induce a significant change in cellular ATP, ADP or AMP levels, direct ones induce conformational changes in the AMPK structural complex because a direct interaction between the modulator and AMPK subunits is needed [6].

Due to the major importance of AMPK in body energy homeostasis, this enzyme has drawn the attention of many researchers as a suitable candidate for the treatment of several metabolic diseases [7].

### 1.2. Metabolic Functions and Physiological Regulation of AMPK

**Figure 2:**
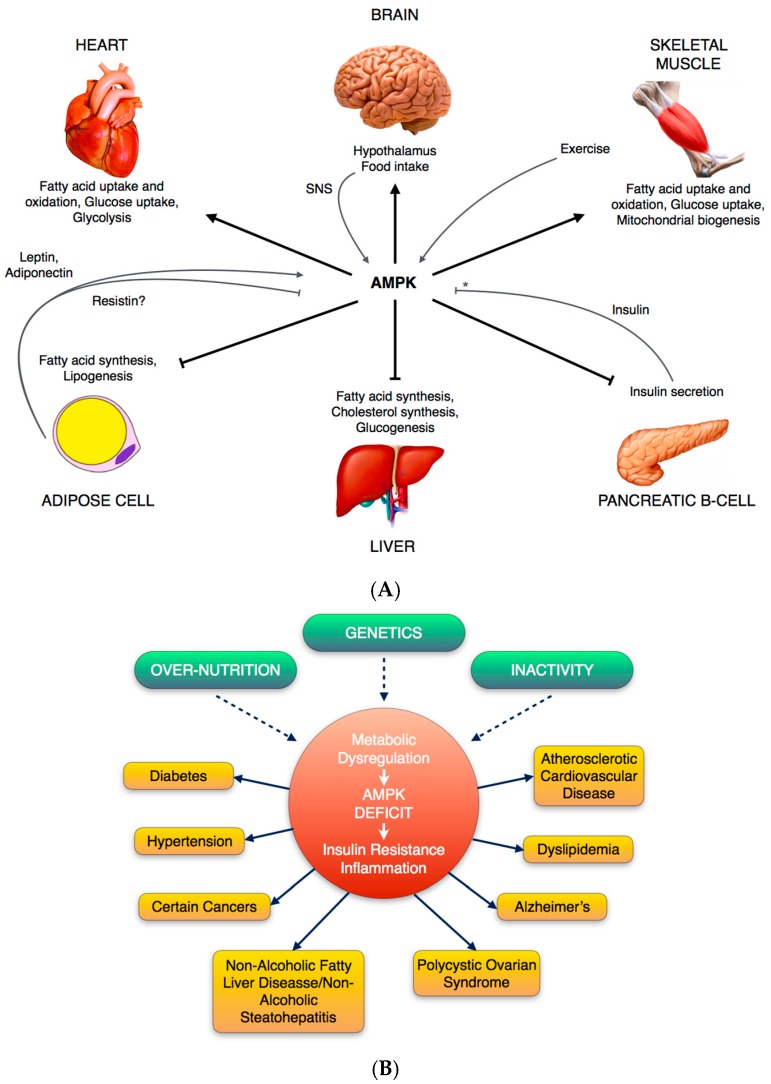
(**A**) Diagram of metabolic functions of AMPK in various tissues; Some key metabolic effects are shown. The adipocyte-derived hormones leptin and adiponectin, as well as exercise, activate (grey arrow) AMPK in skeletal muscle, stimulating fatty acid oxidation. Moreover, leptin’s activation of AMPK in skeletal muscle involves the hypothalamic-sympathetic nervous system (SNS) axis. In hypothalamus, AMPK activity plays a role in regulation of food intake. AMPK inhibits (black T-bar) insulin secretion from pancreatic β cells, and insulin inhibits AMPK activation in ischemic heart and hypothalamus, whilst it has no effect on AMPK in skeletal muscle or adipocytes (*). (**B**) Physiological regulation of AMPK in terms of related diseases and different situations. Overnutrition, inactivity or genetic factors (dashed arrows) can result in a state of dysregulation characterized by inflammation and insulin resistance, that in turn can predispose to one or more of the disorders shown.

When AMPK is activated, a number of signaling pathways are initiated, having effects on lipid, glucose and protein homeostasis. These effects are crucial for the regulation of metabolic events in liver, heart, skeletal muscle, brain and adipose tissue (Figure 2). AMP-activated protein kinase is considered as a fuel sensor for glucose and lipid metabolism.

The therapeutic potential of AMPK activators to treat type 2 diabetes mellitus (T2DM) [8] was suggested when it was discovered that physical exercise activated AMPK in skeletal muscle, which led to increase glucose uptake. Activation of AMPK stimulates glucose transporter type 4 (GLUT4) translocation to the plasma membrane to actively promote increased glucose uptake in skeletal muscle, enabling ATP production through glycolysis. [9]. It is thought that translocation of GLUT4 from skeletal muscle to the plasma membrane is mediated through phosphorylation of TBC1D1 [10] which increases the activity of Rab family G proteins and induces fusion of GLUT4 vesicles with the plasma membrane.

The liver also plays a key role in the maintenance of glucose homeostasis by modulating hepatic glucose during periods of fasting (ATP levels decrease and AMPK is activated as a consequence of energy demand) and feeding. In postprandial situations, hepatic glycogen storage are restored and excess of carbohydrates become triglycerides, originating long-term energy storage. During that process, AMPK is inactivated and anabolic pathways are restored, increasing fatty acid synthesis through ACC activation.

AMPK is then a master metabolic regulator, being responsible for the regulation of anabolic and catabolic processes and whole-body energy homeostasis. Under physiological conditions, the activation of AMPK is related to changes in energy balance. This energy sensor, activated when cellular energy levels are low, results in activation of catabolic processes, and an inactivation of anabolic processes, having, for example, a beneficial effect on glycemia and therefore on diabetes. AMPK also has functions as a regulator of proliferative signals such as mammalian target of rapamycin (mTOR), tuberous sclerosis complex (TSC), ribosomal protein S6 kinase (p70S6) and elongation factor-2, indicating that cancer cell proliferation can be modified via modulating the signaling network through AMPK [11].

Since this kinase is involved in multiple signaling pathways, deregulation of AMPK is associated with a large number of human pathologies, so that the current focus lies in finding AMPK activators. According to this, growing attention is nowadays given to the possible preventive capacity of nutraceutical compounds to prevent illness onset through the modulation of AMPK pathway.

Some foods include several dietary compounds with many health advantages which present a high-grade chance for well-being optimization. Since ancient times, the benefits of foods have been investigated. Epidemiological studies have shown a close relation between the consumption of foods from plants (such as some fruits, vegetables, and grains), and direct health benefits. Glucosinolates, for example, are a group of dietary phytochemicals that have been associated with health benefits, as well as other compounds such as sulfur-containing compounds belonging to the Alliaceae family, terpenoids (carotenoids, monoterpenes, and phytosterols), and, in particular, polyphenols (anthocyanins, flavones, isoflavones, stilbenoids). This wide range of products cannot be truly classified as “food” and a new hybrid term between nutrients and pharmaceuticals, known as “nutraceuticals,” has been used to designate them [12]. A nutraceutical compound is by definition “a food that provides benefits on health in addition to its nutritional content”. Taking that into account, nutraceutical compounds define a precise category, in the frontier between drugs and food.

**Figure 3:**
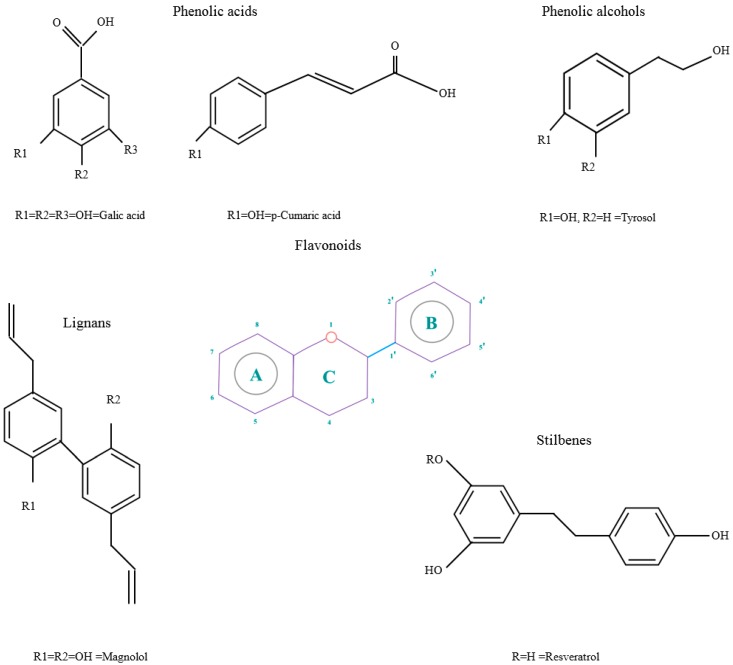
General structure of main groups of polyphenols. Substituents corresponding to concrete structures of some compounds are underlined. Nuclear carbonic atoms corresponding to flavonoids structure are listed.

**Figure 4:**
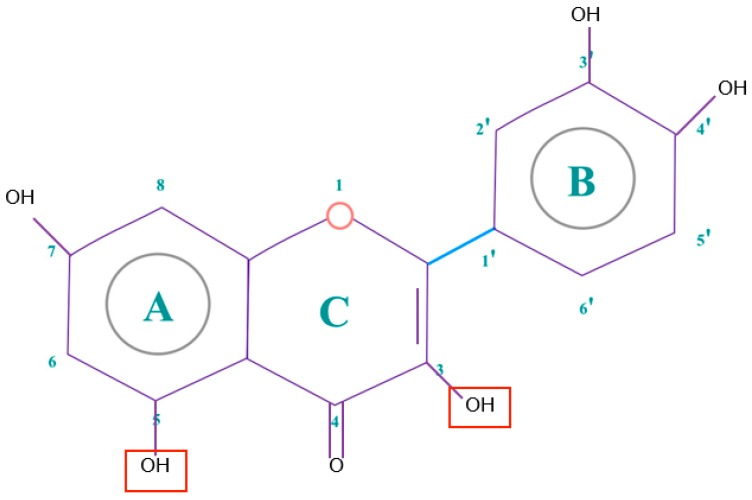
Quercetin structure. Flavonoid that own maximum antioxidant potential. Cathecol structure in the B ring, with hydroxyl groups in 5 and 7 positions, double bond in 2,3 position, conjugated with an 4-oxo group, and 3-OH group in the C ring are represented.

Polyphenols are phytochemical compounds that constitute an important part of human diet. Many different nutraceuticals belonging to this group have been identified in a wide variety of fruits, vegetables and other plant-based foods, such as grains or roots [13]. Chemically, polyphenols contain one or more benzene rings joined to hydroxyl groups that confer the molecule the so-called antioxidant capacity. Based on differences in generic structure of the aromatic rings and the chemical groups attached to them, polyphenols are classified into five different subclasses (Figure 3): flavonoids, phenolic acids (derived from hydroxybenzoic acid or hydroxycinnamic acid), phenolic alcohols, stilbenes and lignans [14]. Flavonoids are the widest group of polyphenols, which in turn, are classified into another five different subgroups: isoflavones, flavonols, anthocyanins, flavones and flavonones [13], quercetin being the best example to explain flavonoids actions concerning to its molecular structure (Figure 4).

Taking into account that flavonoids are the widest group of polyphenols, the structural characteristics in a flavonoid molecule that determine its antioxidant power are:

1. The presence of 3′-4′-*O*-dihydroxy structure on the B aromatic ring (cathecol), which confers more stability and participates in electron delocalization. Studies have reported the relationship between cathecols and its involvement in the inhibition of lipid peroxidation [15].
2. Hydroxyl groups in 5 and 7 positions on the A ring.
3. Double bond localized in 2,3 positions conjugated with a 4-oxo group and the presence of 3-OH in C ring, responsible for the delocaization from B ring.

For maximum antioxidant effectiveness, the presence of all these functions are required. In fact, the absence of some of them induce changes in its activity [16].

Quercetin (3,3′,4′,5,7-pentahydroxyflavone) satisfies the structural requirements that a flavonoid must own to express the maximum antioxidant potential.

It is known worldwide that a healthy lifestyle, including a suitable diet combined with regular exercise is vital to promote well-being and prevent onset of pathology. Metabolic syndrome is a resulting example due to bad habits in lifestyle. This worldwide epidemic threat is characterized by cardio-metabolic risk factors, such as obesity, insulin resistance, hypertension, and dyslipemia. Prevention is therefore, a powerful strategy for an effective medical treatment. Nutraceuticals are a great tool to manage pre-clinical health conditions since they can be included in daily diet to use as illness-onset prophylactics [17]. This review highlights some recent findings related to nutraceutical compounds present in daily diet targeting AMPK pathway, that could have a beneficial effect on health and particularly, on illness onset prevention.

## 2. Nutraceutical Compounds and Cancer

Despite the numerous advances in biomedical and clinical research, cancer remains a leading cause of death worldwide. The progression of cancer is a continual unregulated process resulting in many accumulated abnormalities with gradual progression, eventually leading to cancerous cells growing and dividing in an uncontrolled manner and spreading through tissues and organs [12]. AMPK is proposed as a master controller of cancer, since it plays an important role in the prevention of its development. This protein kinase is responsible for cancer cell proliferation and apoptosis. Natural components, such as polyphenols and in particular flavonoids, target AMPK to induce apoptosis and to avoid cell proliferation [18].

**Figure 5:**
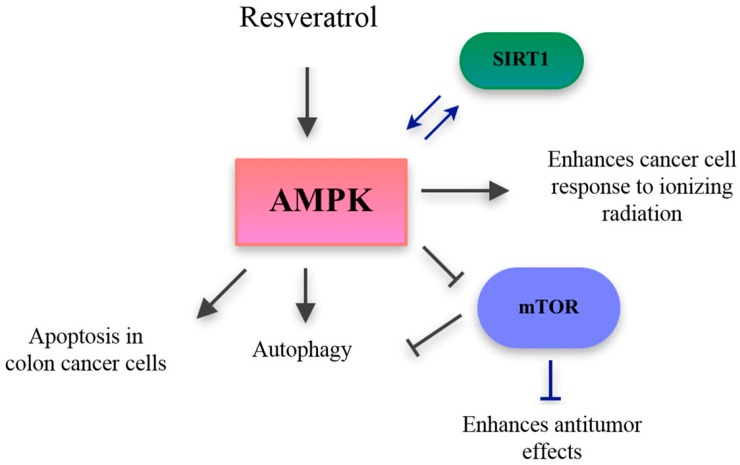
Targeting the AMPK activation through nutraceuticals. Resveratrol activates AMPK leading to apoptosis of colon cancer cells, enhance of cancer cell response to ionizing radiation, and mTOR-dependent and independent autophagy. Resveratrol also activates SIRT1, which improves AMPK activation, leading in turn to downregulation of mTOR. mTOR: mammalian target of rapamycin, SIRT1: Sirtuin-1. Grey and blue arrows indicate activation, T-bars indicate downregulation.

Targeting the mTOR pathway has currently emerged as an interesting tool to control cancer with nutraceuticals, since mTOR promotes tumorigenesis. Modulating the AMPK/mTOR (Figure 5) pathway with phytochemical compounds could be one useful strategy for cancer prevention and control. Some studies performed in human colorectal carcinoma cell line HCT-116 and in vivo, on BALB/C AnN-Foxn1 nude mice treated with HCT-116 cells, have demonstrated the capacity of phenolic acids (caffeic acid; Table 1) to induce apoptosis through the AMPK/mTOR pathway [19]. On the other hand, it has been reported in various studies that grape flavonols, such as quercetin (Table 1), have been involved in cancer protection due to their strong antioxidant and pro-apoptotic effects [20]. Anti-carcinogenic activity of quercetin, present in grapes and other fruits and vegetables, is the most studied activity of flavonoids in cancer. In humans, grape consumption has been associated with reduced breast cancer risk [21] and it has been demonstrated that MCF-7 breast cancer cells treatment with this compound exerted anti-proliferative effects and induced apoptosis through AMPK activation, that in turn modulated apoptotic pathways such as apoptosis signal-regulating kinase 1/p38 MAP Kinase (MAPK) and cyclooxygenase 2 (COX-2), and inhibited the adenosin/threonine (Akt) pathway [18,22,23].

The expression of Bcl-2 and Bax, which are crucial proteins in cell cycle arrest and apoptosis, were modified via AMPK in HT-29 colon cancer cells. In vivo studies were performed to support in vitro evidence by oral administration of quercetin to mice with HT-29 tumor xenografts showing a significant induction of apoptosis and a reduction in tumor volume [24]. Moreover, although flavones are the less common flavonoids found in diet, some, such as luteolin or hispidulin (Table 1), have also shown beneficial effects on cancer cells through AMPK activation. On one side, luteolin causes Reactive Oxygen Species (ROS) release, inducing cell death and inhibiting nuclear factor-κ β (NF-κB) [25]. On the other side, the treatment of human glioblastoma multiform cells and human ovarian cells with hispidulin was able to arrest the cell cycle at the G1 phase, inducing apoptosis [26]. The anticancer properties promoted by flavonols like epillocatechin gallate (EGCG; Table 1) rely on a strong activation of the AMPK pathway by promoting apoptosis in rat hepatoma cells. It has been reported that liver hepatocellular cells (HepG2) treatment with EGCG exerted cell cytotoxicity through stimulation of AMPK while in Hep3B cells the activation of AMPK resulted in a decrease in COX-2 expression [27,28]. According to the evidence, EGCG could be used as an adjuvant in combination with chemotherapy, increasing therapy efficacy in patients.

Isoflavones like genistein (Table 1) have great anticarcinogenic effects through the modulation of AMPK pathway and COX-2 expression. In MCF-7 breast cancer cell lines, genistein has been demonstrated to be directly involved in COX-2 downregulation [29]. In HT29 colon cancer cells genistein was an effective phytochemical to combine with 5-fluouracil, commonly used in colon cancer treatment. This combination led to an increase of ROS and consequently to AMPK activation, decreasing COX-2 expression [30].

Another group of polyphenols of interest are lignans and particularly, magnolol (Table 1), a compound isolated from roots and barks of *Magnolia officinalis* that has recently shown interesting anticancer activity through AMPK modulation [31,32,33]. The treatment of HCT-116 human colon cancer cells with this compound induced apoptosis and exerted anti-proliferative effects in a dose and time dependent manner.

To date, a number of studies have explained the role of AMPK in tumorigenesis [34]. Initially, the connection between AMPK and cancer biology was through the discovery of tumor suppressor LKB1 as a major AMPK upstream kinase [35]. Gene mutations of the *LKB1* gene are responsible for inherited Peutz–Jeghers syndrome, which is characterized by the development of harmartomatous polyps in the intestine [36]. Since then, numerous in vitro and in vivo studies have proposed that AMPK deeply mediates the tumor suppressor effects of LKB1. This is backed by findings that some particular drugs are capable of activating AMPK, such as metformin, phenformin or A-769662, as they are able to delay the onset of tumorigenesis in in vivo models [37] or reduce the rate of cancer risk. [38,39].

However, it is important to note that AMPK functions as either an anti- or pro-tumorigenic regulator depending on the molecular pathway involved.

Tumor suppressor p53 is activated upon oxidative stress and in turn inhibits cell proliferation through particular target genes. Cell proliferation is positively regulated by mTOR, whose action is controlled by the TSC1/TSC2 complex. However, the mechanism by which p53 and oxidative stress negatively control cell growth via TSC1/TSC2/mTOR core is not solidly established. It has been demonstrated that the products of two p53 target genes [40], known as sestrin1 and sestrin2, activate AMPK and target it to phosphorylate TSC2 and, thus inhibit mTOR and cell proliferation.

On the other hand, some authors have reported that mTORC1 [41] and RNA polymerase I transcription initiation factor (TIF-1A) [42], both of which are involved in rapidly growth cells, are controlled by AMPK. For instance, under metabolic stress conditions, tumor cells require AMPK overcome hypoxia and nutrient limitation driven by their uncontrolled proliferation. Normal cells rely less on AMPK than tumor cells, suggesting that sometimes it would be a more effective strategy to inhibit AMPK to combat tumors instead of promoting its activation. Conclusively, the application of AMPK activators in oncology depend on patients circumstances and kind of tumor.

## 3. Nutraceutical Compounds and Cardiovascular Disease

Cardiovascular disease (CVDs), including heart disease and stroke, represent the principal cause of death in western countries [9]. It has been reported that the increased incidence of CVDs by 2030 will lead to more than 23.6 million cardiovascular event-related deaths [43]. This fact has stimulated the research of substances that can improve cardiovascular health. CVD is a worldwide problem that can be prevented by simply developing healthy habits. Among nutraceutical compounds, resveratrol (Table 2) is a very potent antioxidant and anti-inflammatory stilbene present in grapes and red wine that has the ability to up-regulate endothelial NO synthase (eNOS), protecting cardiovascular function through the AMPK pathway [44]. Moreover, AMPK activation is required to attenuate the expression of the intracellular adhesion molecule 1 (ICAM-1), which is involved in atherogenesis [45]. AMPK plays a crucial role in cardiac function, since its inactivation could lead to heart failure. Cardiac dysfunction can be prevented by resveratrol through AMPK modulation, since it has been shown that resveratrol treatment on cardiac function is closely related to its capacity to improve AMPK activity via Sirtuin-1 (SIRT1) activation [46], as shown in vitro on treated cardiomyocytes. A great beneficial effect after treatment with resveratrol was also found in an in vivo model of heart failure of myocardial infarction, enhancing AMPK expression [46]. AMPK activation is also necessary for vascular relaxation, which is mediated by resveratrol, improving endothelium dependent vasodilation [47,48]. AMPK activation has also shown an effect on hypertrophy, inhibiting hypertrophic growth [48]. Some studies have reported that resveratrol may reduce the hypertrophic growth preventing the inhibition of LKB1 and AMPK activity in isolated cardiomyocytes [49,50].

Another group exerting beneficial effects on cardiac function are anthocyanins [51]. In this context, Yang et al. focus on the direct effect of delphinidin-3-glucoside (dp-3-glu) (Table 2) on CVD. Dp-3-glu is the principal pigment present in bilberry fruits and the treatment with this polyphenol shows in both in vitro and in vivo models an inhibition of platelet aggregation and prolonged time required for thrombus formation. It is thought that dp-3-glu surprisingly down-regulates AMPK pathway, attenuating in that way the activation of the cytoplasmic tail of integrin aIIbb3, which is a protein required for clot retraction and thrombus stability. This event inhibits platelet aggregation and thrombus growth, although further studies are necessary to associate anthocyanin consumption and CVD prevention.

Recent reports show that also flavonols, and especially quercetin (Table 2), seem to exert protective effects through AMPK activation [52,53,54]. Quercetin and its metabolites seem to protect endothelial and vascular function. A study performed in mouse endothelial dysfunction-induced with hypochlorous acid (HOCl) showed that quercetin enhanced acetylcholine-mediated endothelial relaxation through the AMPK pathway [55]. These findings were confirmed by inhibiting AMPK with compound C treatment of the aortic rings, which blocked the protective effects of quercetin [56,57]. These results suggest that flavonols targeting AMPK pathway are an interesting tool to manage or prevent cardiac dysfunction onset.

Finally, hydroxytyroxol (HT; Table 2) was also found to ameliorate endothelial functionality, since this phenolic alcohol is able to reduce intracellular ROS levels in porcine pulmonary artery endothelial cells. HT is able to increase the antioxidant activity of catalase through the AMPK/forkhead transcription factor 3a (FOXO3) pathway, proposing HT as an effective phenolic compound for reducing endothelial dysfunction and atherosclerosis [58].

As discussed above, AMPK activators could be deleterious in the treatment of some chronic human pathologies. Recent research have reported that mutations on the *PRKAG2* gene could have cardiovascular adverse effects due to chronic activation of AMPK. For instance, naturally occurring mutations of human γ2 have been associated with enhanced glycogen storage in cardiomyocytes, cardiac hypertrophy, and electrophysiological abnormalities [59]. Then, one should be cautious when AMPK is considered as a therapeutic target.

## 4. Nutraceutical Compounds and Type II Diabetes Mellitus

Type 2 diabetes mellitus (T2DM) is a metabolic disorder characterized by pancreatic β-cell dysfunction, hyperglycemia, and insulin resistance, resulting in glucose and lipid metabolism deregulation. Consequently, low-grade inflammation and oxidative stress arise, leading to micro- and macro-vascular severe complications, such as neuropathy, retinopathy and/or nephropathy, with quality of life considerably diminished [60,61,62]. Development of the disease can be prevented or delayed in people with impaired glucose tolerance by implementing changes in lifestyle, being diet the main modified factor.

The interest of the scientific community in targeting the activation of AMPK pathway as a new treatment for metabolic disorders has arisen as a result of the value of this kinase in managing cellular metabolism and energy control. Phenolic compounds such as quercetin and resveratrol (Table 3), have shown an increased glucose uptake in muscle cells and adipocytes by promoting translocation of GLUT4 via induction of AMPK in vitro [63,64]. Resveratrol has shown important beneficial effects in various in vitro and in vivo studies of human disease models of metabolic disorders [53,65,66,67]. These beneficial effects of stilbenes and more specifically of resveratrol, have been attributed to AMPK as a main target [53,68,69]. When resveratrol activates AMPK, translocation of GLUT4 is mediated, which is essential for the uptake of glucose from the blood to target different organs. Resveratrol is suggested to improve GLUT expression through AMPK phosphorylation in an assay performed in L6 myotubes cells enhancing glucose uptake and, overcoming in a certain way, insulin resistance [70]. Moreover, it have been demonstrated that resveratrol supplementation in db/db mice increased glycolytic activity and fatty acid oxidation [71] and decreased gluconeogenesis in liver, regulating in turn, glucose metabolism in hepatocytes and subsequently, improving hyperglycemia.

Naringin and naringenin (Table 3) are flavonoids present in citrus fruits, some berries, tomatoes and mint, and represent the most common studied compounds among flavonones [72]. In particular, naringin possesses powerful hypoglycemic effects [73]. Some studies have investigated the beneficial effect of naringin in primary hepatocytes exposed to high doses of glucose and in C57BL/6J mice fed with high-fat diet. They concluded that naringin protected against metabolic syndrome onset, up-regulating the AMPK pathway [74]. This activation by phosphorylating AMPK and insulin receptor substrate-1 (IRS-1) led to an improvement in insulin resistance, suppression of gluconeogenesis and an increase in mitochondrial oxidation. Naringenin, which is naringin aglycon, seems to be also involved in lipid and glucose metabolism, stimulating glucose uptake and increasing AMPK phosphorylation in L6 rat skeletal myotubes in a dose- and time-dependent way. Such events emphasize their potential as metabolic disorders preventives [75].

The antidiabetic properties of catechins lie on the protective effect of epigallocatechin gallate (EGCG; Table 3), the major polyphenol present in green tea which seems to possess potentiating activity in glucose utilization [76,77,78,79,80]. EGCG activates AMPK, enhancing insulin signaling pathway by membrane translocation and phosphorylation of IRS-1, improving insulin sensitivity and secretion [81].

Several studies on food containing flavonoids have been reported to mediate blood glucose levels, being helpful for T2DM management. It has been identified that quercetin possesses α-glucosidase inhibitory activity in vitro [84,85]. Indeed, quercetin administration seems to reduce fasting and postprandial glycemia in diabetic mice and rats [86]. In addition to this, quercetin treatments resulted in interesting effects, improving insulin sensitivity in muscle cells through the AMPK pathway, which is involved in cellular glucose uptake by glucose transporter type 4. These findings suggest that quercetin constitutes a nutraceutical compound able to ameliorate insulin resistance in muscle cells through different events linked to AMPK phosphorylation and activation [87]. Phenolic alcohols, such as hydroxytyroxol (Table 3) were also found to increase fatty acid oxidation and to improve insulin sensitivity through AMPK phosphorylation, as shown in 3T3-L1 adipocytes, suggesting its possible involvement in diabetes mellitus management [88]. Berberine and galegine are also natural occurring alkaloids that have been documented to exert several beneficial effects on human health and specially, on diebtes. Both berberine and galegine inhibit mitocondrial function through the inhibition of complex I of the respiratory chain [82] leading to an indirect activation of AMPK due to cessation of mitochondrial ATP production and, an increase in cellular AMP:ATP or ADP/ATP ratios in a similar manner to the biguanides [83]. Berberine has shown some favorable effects on plasma glucose, lipids and glycated haemoglobin (HbA1c) (Table 3) in two clinical trials performed in patients newly diagnosed with type 2 diabetes [83].

## 5. Nutraceutical Compounds and Neurodegenerative Diseases

Neurodegenerative diseases are characterized by progressive degeneration of nerve cells that eventually leads to dementia. Among these diseases, Alzheimer’s (AD), Parkinson’s (PD), Huntington’s (HD) and amyotrophic lateral sclerosis (ALS) can be found. Although they affect different neural populations, they share several characteristics in common. For example, they are characterized by the presence of proteins aggregates in degenerating neurons that certainly derive from defective purification mechanisms including proteosomal dysfunction and lysosomal clearance, and metabolic disturbances, excitotoxicity and oxidative stress are often described [89,90,91]. All of these aspects could participate in the deregulation of AMPK that has been reported to occur in these diseases [92]. In the brain, AMPK acts as a multifunctional metabolic sensor and, depending on the type of stress, cell type and duration of exposure, has a dual role in regulating cell death and survival: its activation incites cell death, while its inhibition produces a protective effect in different models exposed to different stressors [11]. The physiological functions of AMPK remain poorly studied, despite being highly expressed in neurons. Still, AMPK is vital for neuronal survival and genetic ablation of AMPK subunits γ (lochrig mutant, [93]) or β (alicorn mutant, [94]) in studies conducted in *Drosophila*, demonstrating that its genetic ablation induces progressive neurodegeneration.

Given the demographic trend towards an aging population, the prevalence of these neurodegenerative diseases and, therefore, their socioeconomic burden will continue to increase dramatically in the coming decades. Current treatments are symptomatic only; there are no therapies available to cure these diseases [92]. In recent years, there have been considered several alternative approaches to delay the progression of these diseases and nutraceuticals are recently coming under the spotlight [95].

Excitotoxicity has been identified as one of the capital mechanisms involved in the onset and progression of a variety of neurodegenerative pathologies including AD, PD, and HD. Anthocyanins extracted from Korean black beans appear to strongly protect both mouse hippocampal HT22 cells and primary cultures of fetal rat hippocampal neurons against kainic acid (KA) induced excitotoxicity [96]. In fact, the treatment and consumption of isolated anthocyanins was able to inhibit KA-induced phosphorylation of AMPK, attenuate KA-induced deregulation of Ca^2+^ accumulation and ROS, and reduce the KA-induced increase in Bax content and decrease in Bcl-2 content, which slows the rate of apoptosis. These results suggest that anthocyanins may be involved in the neuroprotective mechanism through modulation of the AMPK pathway, but more studies are needed to validate these hypotheses [96].

Recently, a novel mechanism has been proposed whereby, in old mice fed with a high cholesterol diet, quercetin may exert beneficial effects against cholesterol-induced neurotoxicity by activating AMPK and reducing protein phosphatase 2C (PP2C) expression [97]. In these animals, higher levels of brain ROS and carbonyl proteins were detected, along with a decrease in the activity of Cu-Zn superoxide dismutase (Cu-Zn SOD). Treatment with quercetin (Table 4) was able to attenuate all these metabolic disorders, while these effects were attenuated by the administration of compound C. Oral administration of quercetin during a high-cholesterol diet blocked the cholesterol-induced activation of PP2C, promoted the activation of AMPK and subsequently the inactivation of ACC, 3-hydroxy-3-methyl-glutaryl-CoA reductase (HMGCR) and FAS ligand. According to this study, quercetin supplementation decreased the density of CD11b-positive microglia cells (which are the first and main form of active immune defense in the central nervous system) in the hippocampus of these mice and reduced inflammatory markers such as COX-2, inducible nitric oxide synthase (iNOS), interleukin-1 beta (IL-1β), interlukin-6 (IL-6) and tumor necrosis factor α (TNF-α) through suppression of NF-κB in the brains of mice fed high cholesterol. In addition, it down-regulated the expression of the Aβ-converting enzyme 1, which decreased the levels of Aβ and Aβ deposits in the cerebral cortex and hippocampus. Finally, quercetin improved the cognitive deficit of mice fed high cholesterol [97].

Resveratrol (Table 4) seems to exert also a neuroprotective effects through AMPK modulation. For example, its treatment inhibited extracellular signal-regulated kinases (ERK) and mTOR signaling in sensory neurons in a time- and concentration-dependent manner by AMPK activation, providing further evidence for AMPK expression as a novel treatment for acute and chronic pain states [98]. It also protected SH-SY5Y cells against rotenone-induced apoptosis and enhanced α-synuclein degradation in α-synuclein-expressing PC12 cell lines derived from pheocromocytoma of the rat adrenal medulla that promote autophagy via the AMPK/SIRT1 pathway in cellular models of PD [99]. In addition, in Neuro2a cells and primary neurons, resveratrol activated AMPK and stimulated mitochondrial biogenesis in an AMPK-dependent manner, independent of the activation of SIRT1, suggesting that resveratrol could affect homeostasis of neuronal energy [100]. In addition, the activation of the AMPK/SIRT1 axis could be potentially useful in reducing the risk of herpes simplex virus 1 (HSV-1) infection in neurons and the cellular damage associated with reactivation episodes [101].

## 6. Conclusions

Given that AMPK is considered a master regulator of metabolism and has a potential effect in a lot of metabolic pathways, its modulation proposes to be a very important target for the treatment of different diseases. In this review, we have revised several of the most important nutraceutical compounds which have been shown to have an AMPK-mediated therapeutic effect. From these, several have shown an indirect effect on the AMPK activation, so new experiments about knockdown and knockout models will show a more direct perspective of the effect in AMPK activation. In this sense, berberine has been shown to lose effect by knockdown of AMPKα expression [102] with similar results in other compounds such as quercetin [103], proposing the need for this methodology in these studies. However, the effects shown for all these compounds could be evaluated for preventive potential. Could, for example, resveratrol have a preventive effect in the diet with respect to neurodegenerative disorders? Several of the diseases shown in this paper are related to aging, so the effect of these compounds included in the diet or as a supplement must be studied in animal models of age-related diseases in order to know the potential effect in the aging process. On the other hand, an important topic which will need study is the translational effect of doses from animal models to humans. For the in vitro and animal studies cited in the text, the experimental doses could translate into potential human doses depending on the pathology and on the patient requirements. Of course, more studies and clinical trials are needed to establish a therapeutical dose for the treatment of major diseases. To date, it has been documented that natural compounds can be used as prophylactics or to prevent onset of illness, and some experimental research suggests that they could be potential compounds for targeting AMPK. However, several of these compounds (such as resveratrol) require high doses in humans in order to show results, and new capsules with highly-purified doses are being commercialized. Of course, long-term treatment in humans will be needed with these compounds and their presentations.

## Figures and Tables

**Table 1:** List of nutraceutical compounds targeting AMPK pathways in cancer.

NC	Classification	Pathway	Experimental Model	Comments	Nutrient	References
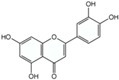 Luteolin	Flavone	- AMPK/NF-κB	- HepG2 Hepatocarcinoma cells - Five-week-old male nude mice	Inhibitory effect on NF-κB Reduces tumor size	Celery, parsley	[14]
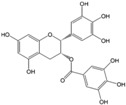 EGCG	Flavonol	- AMPK/p53 expression - AMPK/COX-2	- H4IIE rat cells - p53-positive HepG2 cells - p53-negative Hep3B cells	Stimulation of apoptosis Exerts cell cytotoxicity Decreases COX-2 expression	Fruits, vegetables, tea	[16,17]
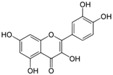 Quercetin	Flavonol	- AMPKα1/COX-2 - AMPKα1/ASK1/p38 pathway	- MCF7 breast cancer cells - HT29 colon cancer cells	Inhibits cell growth Cells cycle arrest Induction of ROS Induction of apoptosis Reduction of tumor volume	Apple, grape, berries, onion, red wine, beans, broccoli, parsley	[12,13,19,22]
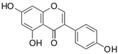 Genistein	Isoflavone	- AMPKα1/COX-2	- MCF-7 breast cancer cells - HT29 colon cancer cells	Reduction COX-2 expression Apoptosis induction	Legumes	[33]
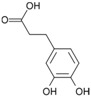 Caffeic acid	Phenolic acid	- PI3K/Akt/AMPK/pmTOR	- Human CRC cells: HTC-116 and BALB/C AnN-Foxn1nude mice	Cell cycle arrest Augmentation of apoptotic pathways	Coffee, argan oil, thyme, sage, spearmint, ceylon cinnamon, star anise	[8]
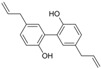 Magnolol	Lignan	- AMPK/pro-apoptotic proteins (p53/Bax)	- HCT 116 Colon cancer cells	Apoptosis induction	Roots and barks of species of *Magnolia officinalis*	[20]

NC: Nutraceutical compound; EGCG: Epigallocatechin gallate; AMPK: Adenosin monophosphate protein kinase; NF-κB: Nuclear factor-κ β; HepG2: Liver hepatocellular cells; COX-2: Cyclooxygenase 2; mTOR: Mammalian target of rapamycin; ROS: Reactive oxygen species; 4EBP1: 4E Binding protein 1; PI3K: Phosphoinositol 3-kinase; Akt: Adenosin/threonine; ASK1: Apoptosis signal-regulating kinase-1; CRC: Colorectal cancer cells.

**Table 2:** List of nutraceutical compounds targeting AMPK pathways in cardiovascular disease.

NC	Classification	Pathway	Experimental Model	Comments	Nutrient	References
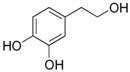 Hydroxytyrosol	Phenolic alcohol	- AMPK/FOXO3	- Porcin pulmonary artery endothelial cells (VECs)	Regulation of antioxidant defense system in VECs	Extra virgin olive oil, leaves from *Olea Europea* L.	[38]
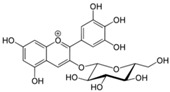 Dp-3-glu	Anthocyanin	- AMPK/integrin aIIbb3	- C57BL/6J mice - Human blood cells who had not taken any platelet medication	Inhibition of both murine and human platelet aggregation Reduction of thrombus growth	Bilberry fruits, cacao, pomegranate	[31]
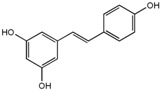 Resveratrol	Stilbene	- AMPK/SIRT1	- In vitro	Prevention of cardiac dysfunction Upregulates eNOS	Skin of grapes, blueberries, raspberries, mulberries and red wine	[24]
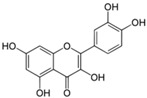 Quercetin	Flavonol	- AMPK/eNOS	- HAECs - Isolated aortic rings from C57BL mice	Induction of eNOS activity Increase of NO production Increase of relaxation	Apple, grape, berries, onion, red wine, beans, broccoli, parsley	[35]

NC: Nutraceutical compound; HT: Hydroxytyrosol; AMPK: Adenosin monophosphate protein kinase; FOXO3: Forkhead transcription factor 3a; VECs: Vascular endothelial cells; Dp-3-glu: Delphinidin-3-glucoside; NO: Nitric oxide; eNOS: endothelial nitric oxide synthase; HAECs: Human aortic endothelial cells.

**Table 3:** List of nutraceutical compounds targeting AMPK pathways in type 2 diabetes mellitus.

NC	Classification	Pathway	Experimental model	Comments	Nutrient	References
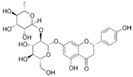 Naringin	Flavonona	- AMPK/IRS-1	- HFD in C57BL/6 mice - Primary hepatocyte cells	Improvement of insulin resistance Stimulation of glucose uptake	Citrus fruits, some berries, tomatoes, mint	[4]
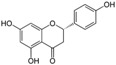 Naringenin	Flavonona	- AMPK/IRS-1	- L6 rat skeletal myotubes	Stimulation of glucose uptake	Citrus fruits, some berries, tomatoes, mint	[4]
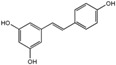 Resveratrol	Stilbene	- AMPK/GLUT4	- L6 myotube cells - db/db mice	Increased glucose uptake in muscle cells and adipocytes Overcome insulin resistance	Skin of grapes, blueberries, raspberries, mulberries and red wine	[33]
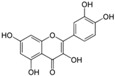 Quercetin	Flavonol	- AMPK/GLUT4	- In vitro	Increased glucose uptake in muscle cells	Apple, grape, berries, onion, red wine, beans, broccoli, parsley	[59,60,61]
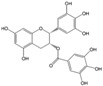 EGCG	Flavonol	- AMPK/IRS-1	- In vitro	Potentiation on the utilization of glucose	Fruits, vegetables, tea	[57]
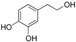 HT	Phenolic alcohol	- AMPK/IRS-1	- 3T3-L1 adipocytes	Insulin sensitivity improvement	Extra virgin olive oil, leaves from *Olea Europea* L.	[63]
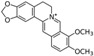 Berberine	Alkaloids	- Indirect activation of AMPK by inhibiting complex I of respiratory chain	- Clinical trial in newly diagnosed type 2 diabetes patients	Favorable effects on glucose, lipids, HbA1c	*Berberis* spp. and other plants	[82,83]
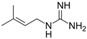 Galegine	Alkaloids	- Indirect activation of AMPK by inhibiting complex I of respiratory chain	- Clinical trial in newly diagnosed type 2 diabetes patients	Favorable effects on glucose, lipids, HbA1c	*Galega officinalis*	[82,83]

NC: Nutraceutical compound; AMPK: Adenosin monophosphate protein kinase; IRS-1: Insulin receptor substrate 1; GLUT4: Glucose transporter type 4; HFD: High fat diet; T2DM: Type 2 diabetes mellitus; EGCG: Epillocatechin gallate; HT: Hydroxytyrosol; HbA1c: Glycated haemoglobin.

**Table 4:** List of nutraceutical compounds targeting AMPK pathways in neurodegenerative disease.

NC	Classification	Pathway	Experimental model	Comments	Nutrient	References
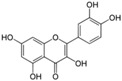 Quercetin	Flavonol	- PP2C/AMPKα/NF-κB	- Intraperitoneal injection in C57BL/6 aged mice	Reduction of neurotoxicity Neuroprotective effect	Apple, berries, onion, red wine, beans, broccoli, parsley, green tea	[72]
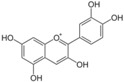 Cyanidin	Anthocyanin	- Regulation of mitochondrial apoptotic (Bax/Bcl-2) pathway	- HT22 cells - Primary hippocampal neuronal cells	Attenuation of ROS accumulation Decrease of apoptosis	Red raspberries, soybean, peach, lychee, red oranges and rice	[71]
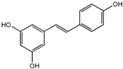 Resveratrol	Stilbene	- AMPK/SIRT1 autophagy	- SH-SY5Y cells - Vero cells - HT22 cells - Rockefeller mice embryos primary cultured neurons	Autophagy-Mediated Neuroprotection Increase viability of HSV-1-infected neurons Inhibit HSV-1 gene expression Inhibit HSV-1 virion progeny productio Reduced neurodegenerative markers	Skin of grapes, blueberries, raspberries, mulberries and red wine	[74,76]

NC: Nutraceutical compound; PP2C: Protein phosphatase 2C; AMPK: Adenosin protein kinase; SIRT1: NAD-dependent deacetylase sirtuin-1; HSV-1: Herpes simplex virus-1; ROS: Reactive oxygen species.

## Abbreviations

ACC1	Acetyl-CoA carboxylase 1
ACC2	Acetyl-CoA carboxylase 2
ADP	Adenosine diphosphate
AMP	Adenosine monophosphate
AMPK	Adenosine monophosphate-activated protein kinase
ATP	Adenosine triphosphate
CaMKKb	Ca^2+^/calmodulin-dependent protein kinase kinase-β
EGCG	Epillocatechin gallate
ERK	Extracellular signal-regulated kinases
GLUT4	Glucose transporter type 4
HbA1c	Glycated haemoglobin
HDAC	Histone deacetylase
HMG-CoA reductase	3-hydroxy-3methyl-glutaryl-coenzyme A reductase
HOCl	Hypochlorous acid
HSV-1	Herpex simplex virus 1
HT	Hydroxytyrosol
ICAM-1	Intracellular adhesion molecule 1
iNOS	Inducible nitric oxide synthase
LKB1	Liver kinase B1
mTOR	Mammalian target of rapamycin
mTORC1	Mammalian target of rapamycin complex 1
NF-κB	Nuclear Factor-κ β
PGC1α	Peroxisome proliferator-activated receptor gamma coactivator 1-α
PP2C	Protein phosphatase 2C
PRKAA1	Protein Kinase AMP-Activated Catalytic Subunit α 1
PRKAA2	Protein Kinase AMP-Activated Catalytic Subunit α 2
PRKAB1	Protein Kinase AMP-Activated Non-Catalytic Subunit β 1
PRKAB2	Protein Kinase AMP-Activated Non-Catalytic Subunit β 2
PRKAG1	Protein Kinase AMP-Activated Non-Catalytic Subunit γ 1
PRKAG2	Protein Kinase AMP-Activated Non-Catalytic Subunit γ 2
PRKAG3	Protein Kinase AMP-Activated Non-Catalytic Subunit γ 3
Raptor	Regulatory-associated protein of mTOR
SIRT1	Sirtuin-1
TBC1D1	Tre-2/Bub2/Cdc16 domain family member
TIF-1A	Transcription initiation factor
TNF-α	Tumor necrosis factor α
TSC2	Tuberous sclerosis complex 2
ULK1	Unc-51-like autophagy activating kinase 1
ZMP	Phosphorylated AICAriboside
